# A predictive model for immunotherapy efficacy in cancer based on dynamic changes of CD8+ T cells: a pan-cancer retrospective study

**DOI:** 10.3389/fimmu.2026.1870840

**Published:** 2026-08-10

**Authors:** Mengyan Xie, Chao Wang, Jun Zhang, Xinming Jing, Pei Ma, Yongqian Shu

**Affiliations:** 1 Department of Oncology, Ruijin Hospital, Shanghai Jiao Tong University School of Medicine, Shanghai, China; 2 Department of Oncology, The First Affiliated Hospital of Nanjing Medical University, Nanjing, China; 3 Shanghai Key Laboratory of Gastric Neoplasms, Shanghai, China; 4 Cancer Center, Daping Hospital & Army Medical Center of PLA, Third Military Medical University, Chongqing, China

**Keywords:** cancer immunotherapy, CD8+ T cells, immune checkpoint inhibitors, lymphocyte subsets, risk stratification

## Abstract

**Objective:**

Immune checkpoint inhibitors (ICIs) have revolutionized cancer treatment, but reliable peripheral blood biomarkers for monitoring treatment response and predicting prognosis remain limited. This study aimed to analyze the dynamic changes of lymphocyte subsets in patients receiving ICI therapy, evaluate their role in treatment response monitoring and prognosis assessment, and develop a practical clinical risk stratification tool.

**Methods:**

A total of 121 patients with malignancies who received ICI therapy and had available lymphocyte subset data were retrospectively enrolled. Peripheral blood lymphocyte subsets and routine blood test data were collected before and after treatment. The associations between changes in these parameters and treatment response as well as progression-free survival (PFS) were analyzed using univariate and multivariate Cox regression models. A risk score model and a simplified clinical scoring system were constructed and validated using time-dependent receiver operating characteristic (ROC) curves and Kaplan-Meier analysis.

**Results:**

Pan-cancer analysis showed that a decrease in CD8+ T cell count after treatment was significantly associated with progressive disease (PD) and inversely correlated with PFS (HR = 0.2308, 95% CI: 0.0875-0.5636). A non-immunotherapy validation cohort further confirmed the immunotherapy-specific nature of CD8+ T cell dynamics. Multivariate Cox analysis identified decreased CD8+ T cell count, elevated neutrophil-to-lymphocyte ratio (NLR), multiple lines of therapy, and specific cancer types (hepatopancreatobiliary malignancies) as independent unfavorable prognostic factors. Time-dependent area under the curve (AUC) values at 2.5, 3.5, and 5.7 months were 0.727, 0.827, and 0.853, respectively, indicating good predictive performance. The risk score based on these variables stratified patients into low-, medium-, and high-risk groups (median PFS: not reached, not reached, and 4.5 months, respectively; p<0.001). A simplified clinical scoring system also effectively distinguished different prognostic groups (median PFS: not reached, 6.2 months, and 4.3 months, respectively; p<0.001).

**Conclusions:**

The dynamic change in CD8+ T cell count before and after treatment is an independent predictor of PFS in patients receiving ICI therapy and exhibits immunotherapy specificity. The proposed risk stratification tool, incorporating CD8+ T cell dynamics, NLR change, and key clinical variables, provides a simple and effective approach for prognostic assessment and may facilitate individualized treatment decision-making in clinical practice.

## 1 Introduction

The human immune system acts as an exogenous tumor suppressor that disrupts developing tumors or inhibits their growth, thereby preventing tumor initiation and progression. However, cancer can still occur in immunocompetent individuals, partly because cancers can induce immune suppression. This immunosuppression is primarily mediated by cytotoxic T-lymphocyte-associated antigen-4 (CTLA-4) and programmed cell death protein-1 (PD-1). Consequently, the advent of immune checkpoint inhibitors (ICIs) represents a major breakthrough in oncology, offering long-term survival benefits for patients with various malignancies (1, 2). ICIs targeting PD-1/programmed death-ligand 1 (PD-L1) and CTLA-4 have established standard treatment positions in multiple cancer types, including lung cancer, melanoma, head and neck squamous cell carcinoma, and renal cell carcinoma (3–7), and more clinical trials are underway to further expand their indications.

Currently, commonly used predictive biomarkers for ICI efficacy include PD-L1 expression level (assessed by tumor proportion score (TPS) or combined positive score (CPS)), tumor mutational burden (TMB), and microsatellite instability (MSI) (8–10). Nevertheless, tumor heterogeneity and the dynamic evolution during treatment limit the ability of a single tissue biopsy to fully capture the immune status. Moreover, due to the potential occurrence of “pseudoprogression” induced by immunotherapy (11, 12), the imaging-based RECIST 1.1 criteria are insufficient to accurately determine the true disease status in patients receiving immunotherapy. Although iRECIST criteria have been recommended in immunotherapy clinical trials to address this limitation (13), simple and effective predictive factors for treatment response and prognosis remain lacking in clinical practice to assist in disease assessment and treatment guidance. Therefore, the development of novel, non-invasive, dynamically monitorable, and immunotherapy-specific biomarkers is of significant clinical importance.

Peripheral blood, as a key source for”liquid biopsy”, offers distinct advantages including easy accessibility, repeatable sampling, and real-time reflection of systemic immune status. Lymphocyte subset analysis, a well-established flow cytometry-based assay, is widely used in clinical practice. Among these subsets, CD8+ cytotoxic T lymphocytes are the core effector cells mediating anti-tumor immune responses. Studies have shown that functional exhaustion of CD8+ T cells in the tumor microenvironment is a crucial mechanism underlying immunotherapy resistance (14, 15). ICI therapy can enhance the cytotoxic effect of natural killer (NK) cells and prolong their survival (16). Regulatory T cells (Tregs) suppress anti-tumor immune responses, and abundant Treg infiltration in tumor tissues is generally associated with poor prognosis, whereas anti-CTLA-4 antibodies may attenuate their suppressive effects by depleting effector Tregs (17). However, whether dynamic changes in peripheral blood lymphocytes reflect the status of anti-tumor immune responses and can serve as biomarkers for monitoring ICI efficacy and assessing prognosis currently lacks sufficient evidence-based medical support.

In recent years, several studies have investigated the relationship between dynamic changes in peripheral blood immune cells and immunotherapy outcomes. Fiala et al. (18) found that early changes in neutrophil-to-lymphocyte ratio (NLR) (ΔNLR≥2) after one month of nivolumab treatment were independently associated with poorer progression-free survival (PFS) and overall survival (OS) in patients with metastatic renal cell carcinoma. In head and neck squamous cell carcinoma, pre-treatment NLR, platelet-to-lymphocyte ratio (PLR), and monocyte-to-lymphocyte ratio (MLR) were significantly associated with response to immunotherapy, and an elevated post-treatment NLR indicated poor prognosis (19). Another study, utilizing single-cell technology, demonstrated that dynamic changes in the polyfunctionality of peripheral blood CD8+ T cells could predict immunotherapy response and clinical outcomes in lung cancer patients (20). These findings suggest that dynamic changes in peripheral blood immune cells hold potential as biomarkers for immunotherapy. However, previous studies have mostly been confined to single cancer types, and comprehensive comparative analyses across different lymphocyte subsets (such as CD4+ T cells, CD8+ T cells, Tregs, B cells, etc.) are still lacking.

Based on the above background, this study aimed to systematically evaluate the dynamic changes in peripheral blood lymphocyte subsets (including cells with fluorescently-labeled antibody of: CD3+, CD3+CD4+, CD3+CD8+, CD19+, CD16+CD56+, CD4+CD25+FoxP3+) before and after immunotherapy, and to analyze their associations with treatment efficacy and prognosis. We first explored potential predictive indicators in a lung cancer subgroup, subsequently validate them in a pan-cancer cohort, and confirm their specificity through a non-immunotherapy cohort. On this basis, we integrated clinical variables to establish a simple and feasible risk stratification tool, thereby providing a reference for individualized management of patients receiving ICI therapy.

## 2 Materials and methods

### 2.1 Study population

**Figure 1:**
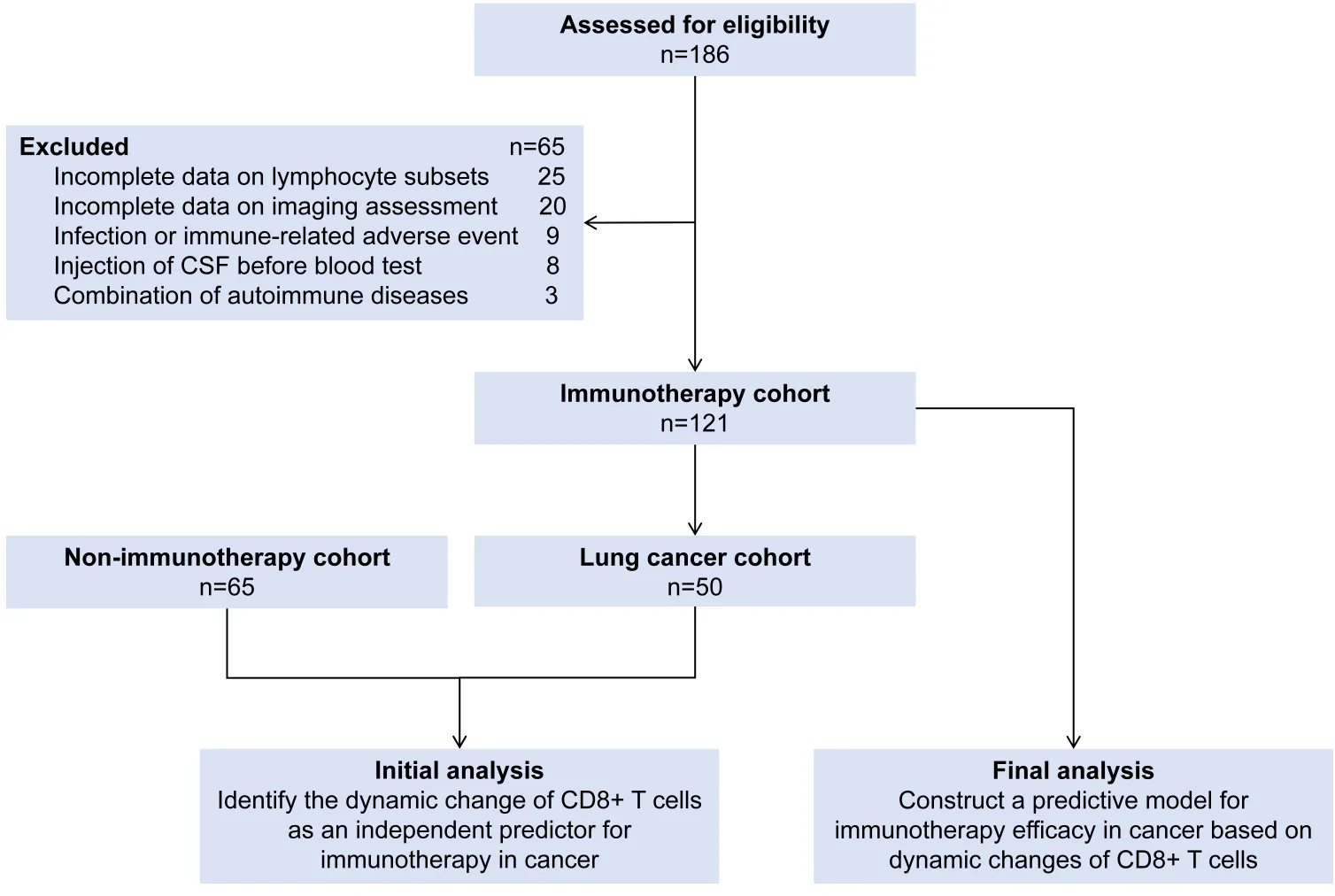
Study profile of patients’ enrollment and analysis.

This study enrolled patients (n=186) with malignant solid tumors who received ICI therapy at Jiangsu Province Hospital (The First Affiliated Hospital of Nanjing Medical University) from November 1, 2020, to October 31, 2021. The main inclusion criteria were: (1) pathologically confirmed malignancy; (2) treatment with PD-1/PD-L1 or CTLA-4 inhibitors as monotherapy or combination therapy; (3) locally advanced or distant metastasis; (4) presence of at least one measurable lesion; (5) lymphocyte subset testing performed during treatment; (6) imaging evaluation of disease status before and after treatment. Exclusion criteria included: (1) concurrent autoimmune diseases or long-term use of immunosuppressants; (2) blood transfusion or colony-stimulating factor (CSF) therapy within 4 weeks before treatment; (3) incomplete clinical data (patients’ basic information, follow-up data, lymphocyte subset data, NLR, imaging evaluation data); (4) active infection or immune-related adverse events (irAEs) at the time of lymphocyte subset testing (**Figure 1**).

After applying the inclusion and exclusion criteria, 121 patients were finally included. Among them, there were 50 patients with lung cancer (non-small cell lung cancer and small cell lung cancer), 27 with upper gastrointestinal malignancies (esophageal cancer, gastric cancer), 11 with genitourinary malignancies (renal cancer, bladder cancer, prostate cancer, cervical cancer, ovarian cancer, urachal carcinoma, testicular cancer), 8 with hepatopancreatobiliary malignancies (liver cancer, pancreatic cancer, cholangiocarcinoma), 6 with malignant melanoma, and 19 with other malignancies (colorectal cancer, breast cancer, cutaneous squamous cell carcinoma, malignant mesothelioma, sarcoma, neuroendocrine carcinoma, nasopharyngeal carcinoma, tonsillar carcinoma).

To verify the immunotherapy-specific association of CD8+ T cell changes, an additional cohort of patients with malignancies receiving non-immunotherapy (chemotherapy and/or targeted therapy, n=65) was included as a validation cohort.

This study was conducted in accordance with the Declaration of Helsinki. The study protocol was approved by the Institutional Review Board (IRB) of Jiangsu Province Hospital (The First Affiliated Hospital of Nanjing Medical University) (Approval No.: 2020-SR-150). Due to the retrospective nature of the study, all data were derived from routine clinical practice without additional intervention or risk to patients, and the IRB granted a waiver of informed consent. All patient information was anonymized during the study to protect personal privacy.

### 2.2 Laboratory tests

#### 2.2.1 Peripheral blood lymphocyte subset analysis

Peripheral venous blood (2–3 mL) was collected from patients before the start of immunotherapy and during treatment (before each immunotherapy cycle) into EDTA anticoagulant tubes. The absolute counts and percentages of the following lymphocyte subsets were measured by flow cytometry: CD3+ T cells, CD3+CD4+ T cells, CD3+CD8+ T cells, CD4/CD8 ratio, CD19+ B cells, CD16+CD56+ NK cells, and CD4+CD25+FoxP3+ Treg cells.

The assay was performed using monoclonal antibody direct immunofluorescence labeling with the MultiTEST IMK kit (BD Biosciences) for absolute counting. All samples were tested within 4 hours of collection. The following dynamic change indicators were calculated: baseline value, last value, and change (last minus baseline).

#### 2.2.2 Routine blood tests and inflammatory markers

Peripheral blood was collected simultaneously with lymphocyte subset testing, and neutrophil, lymphocyte counts were measured using an automated hematology analyzer. NLR were calculated: NLR = neutrophil count/lymphocyte count. Changes (increase or decrease) of NLR before and after treatment were also calculated.

### 2.3 Efficacy assessment

#### 2.3.1 Imaging evaluation

All patients underwent baseline imaging (contrast-enhanced CT or MRI of the chest, abdomen, and pelvis) within 4 weeks before immunotherapy, and then imaging was repeated every 8–12 weeks (3–4 treatment cycles) until disease progression, death, loss to follow-up, or study end. If clinical symptoms or other parameters strongly suggested disease changes, additional imaging was performed for disease assessment after confirmation by the attending physician. The best treatment response was evaluated according to RECIST version 1.1:

- Complete response (CR): disappearance of all target lesions
- Partial response (PR): ≥30% decrease in the sum of diameters of target lesions
- Stable disease (SD): neither sufficient shrinkage to qualify for PR nor sufficient increase to qualify for PD
- Progressive disease (PD): ≥20% increase in the sum of diameters of target lesions or appearance of new lesions

Patients were divided into three groups: CR/PR, SD, and PD.

#### 2.3.2 Follow-up and definition of PFS

Follow-up was conducted through outpatient visits, hospital records, and telephone interviews. The starting point of follow-up was the date of first ICI treatment, and the endpoints were PD, death, or the last follow-up date. PFS was defined as the time from first treatment to the first imaging-confirmed disease progression, death from any cause, or loss to follow-up. For patients who had not experienced disease progression, death, or loss to follow-up by the cutoff date (February 28, 2023), the last follow-up date was used for censoring.

### 2.4 Statistical methods

#### 2.4.1 Software platform

All statistical analyses were performed using GraphPad Prism (version 11.0.0) and R software (version 4.5.3) with relevant extension packages (forestplot, tidyverse, readxl, survival, timeROC, ggplot2, rms, regplot, MASS, dplyr, survminer, shiny, shinythemes, rsconnect). The detailed R code can be found in **Supplementary Materials**. Two-sided tests were used, and *p* < 0.05 was considered statistically significant.

#### 2.4.2 Univariate analysis

In the lung cancer subgroup (n=50), we first analyzed the relationships between baseline values, last values, and changes (increase/decrease) of each lymphocyte subset (percentages/counts) with treatment response and PFS. Tukey’s multiple comparisons test was used to compare differences in continuous variables among different response groups. Kaplan-Meier curves were plotted for PFS among different patient groups, and survival differences were compared using the log-rank test. The above analyses were repeated in the pan-cancer cohort (n=121) and the non-immunotherapy validation cohort (n=65) to confirm generalizability and specificity.

#### 2.4.3 Cox proportional hazards regression model

Variables included in the multivariate Cox regression model were: age, sex, ECOG score, line of therapy, cancer type, CD4/CD8 change, CD8+ T cell change, and NLR change. Coding for cancer types and model variables is shown in **Tables 1** and **2**. Hazard ratios (HRs) and their 95% confidence intervals (CIs) were calculated. Model goodness-of-fit was assessed using the Akaike information criterion (AIC), and discrimination was assessed using Harrell’s C-index.

**Table 1:** Cancer type classification and coding.

Cancer type	Specific cancers	Code
Lung cancer	Non-small cell lung cancer, Small cell lung cancer	0
Upper gastrointestinal malignancies	Esophageal cancer, Gastric cancer	1
Genitourinary malignancies	Renal cancer, Bladder cancer, Prostate cancer, Cervical cancer, Ovarian cancer, Urachal carcinoma, Testicular cancer	2
Hepatopancreatobiliary malignancies	Liver cancer, Pancreatic cancer, Cholangiocarcinoma	3
Malignant melanoma	–	4
Others	Colorectal cancer, Breast cancer, Cutaneous squamous cell carcinoma, Malignant mesothelioma, Sarcoma, Neuroendocrine carcinoma, Nasopharyngeal carcinoma, Tonsillar carcinoma	5

**Table 2:** Variable coding for multivariate cox regression.

Variable	Coding	Reference to
Age	Continuous	–
Sex	Female = 0, Male = 1	0
ECOG	0-1 = 0, ≥2 = 1	0
Therapy Line	First-line = 1, Second-line = 2, Third-line or above= 3	1
Cancer Type	See **Table 1**	0
CD4/CD8 Change	Increase = 0, Decrease = 1	0
CD8+ T cell Change	Increase = 0, Decrease = 1	1
NLR Change	Increase = 0, Decrease = 1	0

#### 2.4.4 Time-dependent ROC analysis

To evaluate the predictive accuracy of the model at different time points, time-dependent receiver operating characteristic (ROC) analysis was performed. The quartiles of PFS event times (2.5 months, 3.5 months, and 5.7 months) were selected as evaluation time points, and the area under the curve (AUC) at each time point was calculated. Due to the limited number of events (n=34), confidence intervals for the AUCs were not estimated.

#### 2.4.5 Risk score construction

##### 2.4.5.1 Risk score-based risk stratification

A linear risk score formula was constructed based on the regression coefficients (β) from the multivariate Cox regression model:

Risk Score = -0.008874×Age + (-0.01482)×Sex[1] + (-0.1993)×ECOG[1] + 0.6255×line[2] + 0.704×line[3] + 0.3053×cancer_type[1] + 1.257×cancer_type[2] + 2.56×cancer_type[3] + 1.312×cancer_type[4] + 1.149×cancer_type[5] + (-0.4928)×CD4/CD8_change[1] + (-1.466)×CD8_change[0] + (-0.8289)×NLR_change[1].

Patients were divided into three groups based on the tertiles of the Risk Score: low-risk group (Risk Score < -2.093), medium-risk group (-2.093 ≤ Risk Score < -0.846), and high-risk group (Risk Score ≥ -0.846). Kaplan-Meier curves and log-rank tests were used to compare PFS among the three groups, and 3-, 6-, and 12-month PFS rates were calculated.

An interactive web-based calculator for the Risk Score was developed using the Shiny R package and is available at: *https://xmy13181.shinyapps.io/CALCULATOR/*. Clinicians can input patient variables online to obtain the Risk Score and risk stratification in real time.

##### 2.4.5.2 Simplified clinical scoring system

To facilitate clinical application, the categorical variables that were statistically significant in the multivariate Cox regression were simplified and assigned points as follows:

- CD8+ T cell change: increase = 0 points, decrease = 2 points
- NLR change: decrease = 0 points, increase = 1 point
- ECOG score: 0 = 0 points, ≥2 = 1 point
- Line of therapy: first-line = 0 points, second-line = 1 point, third-line or above = 2 points

The total score for each patient ranged from 0 to 5. According to the score distribution, patients were divided into three groups: low-risk (0–1 points), medium-risk (2–3 points), and high-risk (4–5 points). Kaplan-Meier curves and log-rank tests were used to compare PFS among the three groups, and median PFS as well as 3-, 6-, and 12-month PFS rates were calculated.

##### 2.4.5.3 Nomogram

Due to the limited sample size and small number of events (n=34), a nomogram constructed based on a stepwise regression-selected multivariate Cox model showed poor calibration (the predicted probabilities deviated considerably from the actual observed probabilities). Therefore, the nomogram was not used as the primary prediction tool, and the risk score and simplified scoring system were presented as the final models.

## 3 Results

### 3.1 Baseline characteristics of patients

A total of 121 patients with malignancies treated with ICIs were included. The median age was 63 years (range: 32–84 years). There were 76 male patients (62.8%) and 45 female patients (37.2%). ECOG scores were 0–1 in 108 patients (89.3%) and ≥2 in 13 patients (10.7%). Regarding lines of therapy, 81 patients (67.0%) received first-line treatment, 17 (14.0%) second-line, and 23 (19.0%) third-line or above. The most common cancer type was lung cancer (50 cases, 41.3%), followed by upper gastrointestinal malignancies (27 cases, 22.3%), genitourinary malignancies (11 cases, 9.1%), hepatopancreatobiliary malignancies (8 cases, 6.6%), malignant melanoma (6 cases, 5.0%), and others (19 cases, 15.7%). Distant metastasis was present in 78 patients (64.5%), and absent in 43 (35.5%). All patients received PD-1/PD-L1 inhibitors, with 111 (91.7%) receiving PD-1 inhibitors and 10 (8.3%) receiving PD-L1 inhibitors. Additionally, 65 patients with malignancies receiving non-immunotherapy (chemotherapy/targeted therapy alone or in combination) were included. Baseline characteristics of all patients are shown in **Table 3**. As of the follow-up cutoff date (February 28, 2023), the median follow-up time was 5.3 months (95% CI: 4.3-6.0 months; range: 0.8-35.5 months). A total of 34 progression or death events (28.1%) occurred, and 87 patients (71.9%) were censored.

**Table 3:** Baseline clinical characteristics of patients.

Clinical characteristics	Immunotherapy cohort (n=121)	Non-immunotherapy cohort (n=65)
Age (years), median(IQR)	63 (55, 73)	62 (51, 74)
Gender, n (%)		
Male	76 (62.8%)	40 (61.5%)
Female	45 (37.2%)	25 (38.5%)
Cancer type		
Lung cancer	50 (41.3%)	25 (38.5%)
Upper gastrointestinal malignancies	27 (22.3%)	24 (36.9%)
Genitourinary malignancies	11 (9.1%)	–
Hepatopancreatobiliary malignancies	8 (6.6%)	–
Malignant melanoma	6 (5.0%)	–
Others	19 (15.7%)	16 (24.6%)
Distant metastasis, n (%)		
M0	43 (35.5%)	16 (24.6%)
M1	78 (64.5%)	49 (75.4%)
Therapy line		
First-line	81 (67.0%)	52 (80.0%)
Second-line	17 (14.0%)	10 (15.4%)
Third-line or above	23 (19.0%)	3 (4.6%)
Immunotherapy regimen, n (%)		
PD-1 inhibitor	111 (91.7%)	–
PD-L1 inhibitor	10 (8.3%)	–
ECOG, n (%)		
0-1	108 (89.3%)	60 (92.3%)
≥2	13 (10.7%)	5 (7.7%)

### 3.2 Relationship between dynamic changes in lymphocyte subsets and treatment response

#### 3.2.1 Lung cancer subgroup analysis

**Figure 2:**
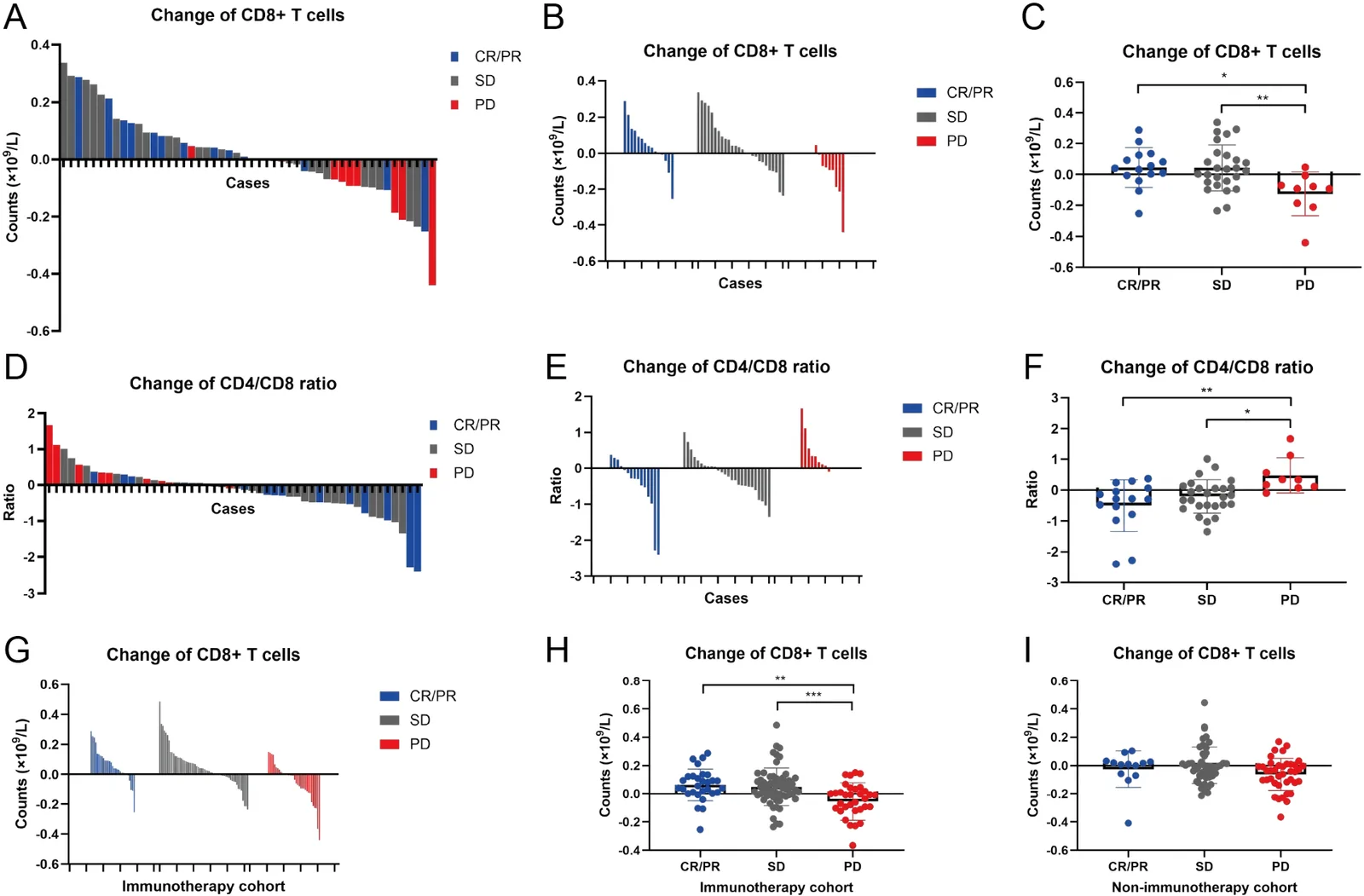
Associations of changes in peripheral blood CD8+ T cell count and CD4/CD8 ratio with treatment response. Waterfall plots **(A, B, D, E, G)** show individual patient changes. Bar graphs **(C, F, H, I)** show group comparisons (mean ± SEM). The unit for the change in cell count is (×10^9^/L)and the CD4/CD8 ratio has no unit. **(A–C)** Lung cancer subgroup (n=50): Waterfall plot showing individual patient changes in CD8+ T cell count. PD was associated with decreased CD8+ T cells (CR/PR vs. PD, *p* = 0.0174; SD vs. PD, *p* = 0.0097). **(D–F)** Lung cancer subgroup (n=50): Waterfall plot showing individual patient changes in CD4/CD8 ratio. PD was associated with increased CD4/CD8 ratio (CR/PR vs. PD, *p* = 0.0023; SD vs. PD, *p* = 0.0249). **(G, H)** Pan-cancer immunotherapy cohort (n=121): Waterfall plot showing individual patient changes in CD8+ T cell count. PD was associated with decreased CD8+ T cells (CR/PR vs. PD, *p* = 0.0013; SD vs. PD, *p* = 0.0008). **(I)** Non-immunotherapy cohort (n=65): The change in CD8+ T cell count was not significantly associated with treatment response (*p* = 0.0561). *p < 0.05, **p < 0.01, ***p < 0.001.

We first explored the relationships between pre-treatment lymphocyte subset counts/percentages, on-treatment lymphocyte subset counts/percentages at the time of imaging assessment, and changes in these parameters between pre- and post-treatment with treatment response in lung cancer patients. Significant differences were found among the CR/PR, SD, and PD groups for changes in absolute CD8+ T cell count (P = 0.0087) and CD4/CD8 ratio (*p* = 0.0033). Compared with patients achieving CR/PR or SD, those with PD had a significant decrease in post-treatment absolute CD8+ T cell count (mean change ×10^9^/L: CR/PR vs. PD 0.1706 ± 0.05979, *p* = 0.0174; SD vs. PD 0.1685 ± 0.05485, *p* = 0.0097) and a significant increase in CD4/CD8 ratio (mean change: CR/PR vs. PD -0.9784 ± 0.2733, *p* = 0.0023; SD vs. PD -0.6794 ± 0.2507, *p* = 0.0249) (**Figures 2A–F**). Pre-treatment lymphocyte subset counts/percentages and on-treatment values at imaging assessment were not associated with treatment response. Changes in the percentages of lymphocyte subsets, as well as changes in total CD3+ T cells, CD4+ T cells, B cells, NK cells, and Treg cells, showed no statistically significant differences among response groups (**Supplementary Figures 1**–**3**).

#### 3.2.2 Pan-cancer cohort analysis

After expanding the analysis to all 121 patients across cancer types, the PD group still showed a significant decrease in post-treatment CD8+ T cell count compared with the CR/PR and SD groups (mean change: CR/PR vs. PD 0.1181 ± 0.0327, *p* = 0.0013; SD vs. PD 0.1047 ± 0.02794, *p* = 0.0008) (**Figures 2G, H**). Notably, changes in CD4/CD8 ratio did not reach statistical significance between the PD group and the SD group in the pan-cancer analysis (mean change: CR/PR vs. PD -0.3886 ± 0.1580, *p* = 0.0405; SD vs. PD -0.2491 ± 0.1350, *p* = 0.1597), and the changes in CD4/CD8 ratio were not significantly associated with response in the 71 patients with other cancer types (*p* = 0.8958) (**Supplementary Figures 4A-C**).

#### 3.2.3 Non-immunotherapy validation cohort

To verify the immunotherapy-specific association of CD8+ T cell changes, we included a cohort of patients with malignancies receiving non-immunotherapy (chemotherapy and/or targeted therapy alone, n=65). In this cohort, change in CD8+ T cell count before and after treatment was not significantly associated with treatment response or prognosis (*p* = 0.0561) (**Figure 2I**), suggesting that CD8+ T cell dynamics are specific to immunotherapy.

### 3.3 Survival analysis

**Figure 3:**
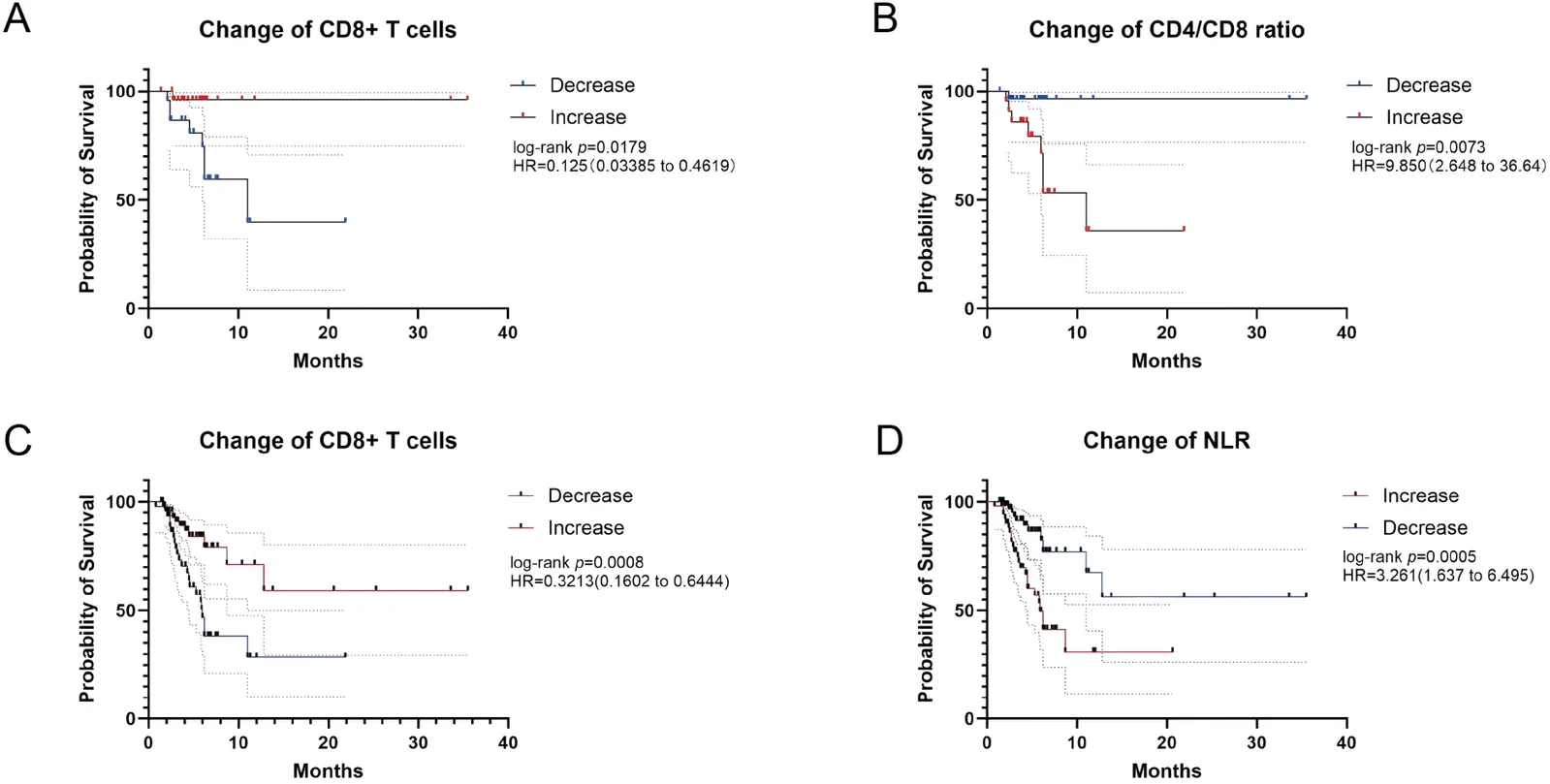
Kaplan-Meier curves for PFS according to dynamic changes in immune-related parameters. **(A)** Lung cancer subgroup (n=50): CD8+ T cell change; increase vs. decrease: median PFS not reached vs. 11.0 months, *p* = 0.0179. **(B)** Lung cancer subgroup (n=50): CD4/CD8 ratio change; increase vs. decrease: 11.0 months vs. not reached, *p* = 0.0073. **(C)** Pan-cancer cohort (n=121): CD8+ T cell change; increase vs. decrease: not reached vs. 6.0 months, *p* = 0.0008. **(D)** Pan-cancer cohort (n=121): NLR change; increase vs. decrease: 5.9 months vs. not reached, *p* = 0.0005. Tick marks indicate censored data.

Kaplan-Meier survival analysis showed that in the lung cancer subgroup, patients with an increased post-treatment CD8+ T cell count had significantly longer median PFS than those with a decreased count (not reached vs. 11.0 months, log-rank *p* = 0.0179) (**Figure 3A**). Patients with an increased CD4/CD8 ratio had significantly worse PFS than those with a decreased or stable ratio (11.0 months vs. not reached, log-rank *p* = 0.0073) (**Figure 3B**).

In the pan-cancer cohort, the CD8+ T cell count increase group had significantly better median PFS than the decrease group (not reached vs. 6.0 months, log-rank *p* = 0.0008) (**Figure 3C**), whereas the correlation between CD4/CD8 ratio change and PFS did not reach statistical significance (log-rank *p* = 0.1228) (**Supplementary Figure 4D**).

Based on literature reports regarding the prognostic value of NLR in specific cancer types receiving immunotherapy, we analyzed the relationships between baseline NLR and changes of NLR with prognosis in our cohort. The results indicated that the NLR increase group had significantly shorter median PFS than the NLR decrease group (5.9 months vs. not reached, log-rank *p* = 0.0005) (**Figure 3D**). Baseline NLR was not significantly associated with PFS (log-rank *p* = 0.1461) (**Supplementary Figure 4E**).

### 3.4 Multivariate Cox regression analysis

**Figure 4:**
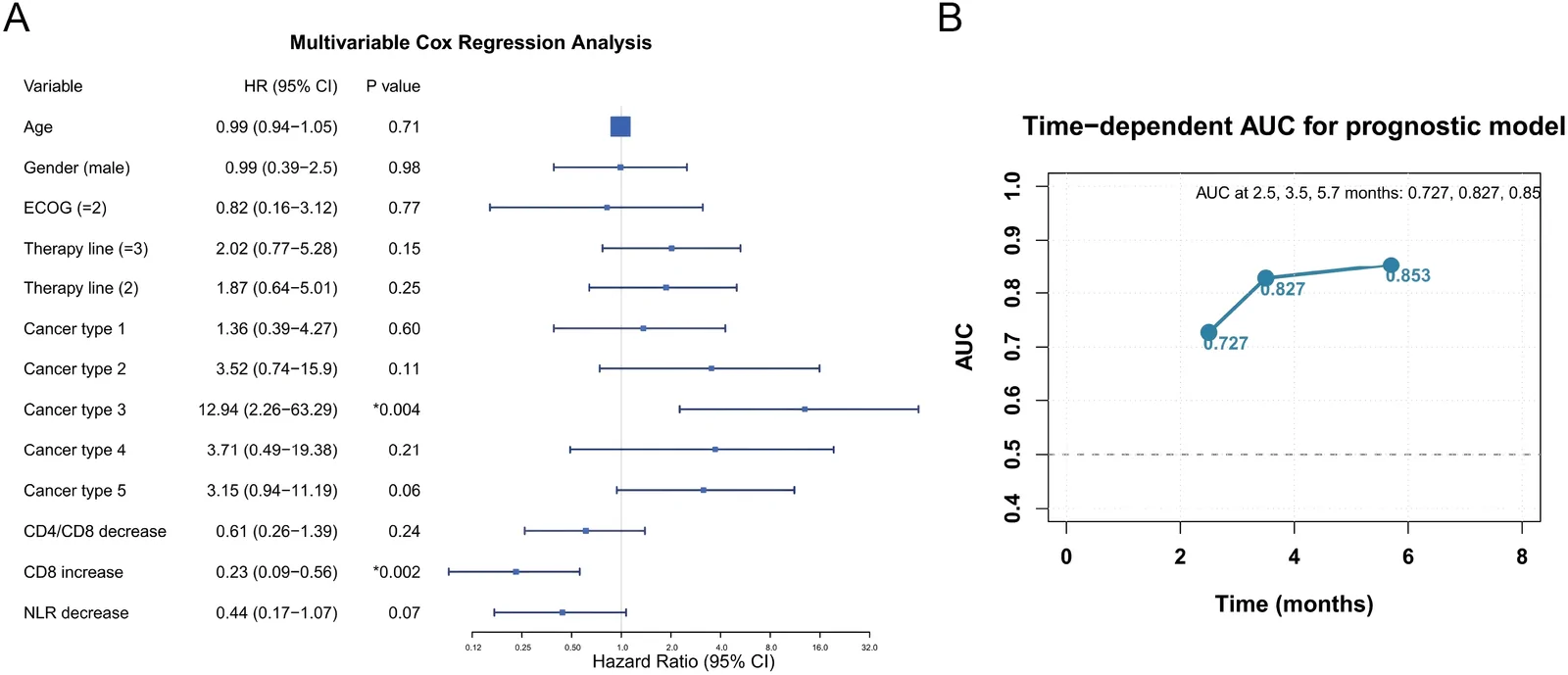
Forest plot and time-dependent ROC curves of multivariate Cox regression analysis. **(A)** This forest plot displays the HRs and 95% CIs of factors associated with PFS in patients receiving ICI therapy. A multivariate Cox proportional hazards regression model was used, incorporating the following variables: age, sex, ECOG score, line of therapy, cancer type (reference: Cancer type 0= Lung cancer), change in CD4/CD8 ratio, change in CD8+ T cell count, and change in NLR. Detailed coding for each variable is provided in **Table 1** and **Table 2**. **(B)** This figure presents the predictive accuracy of the multivariate Cox regression model for PFS at different time points in patients receiving ICI therapy. The three quartiles of PFS event times (2.5 months, 3.5 months, and 5.7 months) were selected as evaluation time points, and time-dependent ROC curves were generated. The point estimates of AUC were 0.727 at 2.5 months, 0.827 at 3.5 months, and 0.853 at 5.7 months. Confidence intervals for the AUCs were not estimated due to the limited number of events (n=34) in this study.

Multivariate Cox regression analysis was performed with age, sex, ECOG score, line of therapy, cancer type, CD4/CD8 change, CD8+ T cell change, and NLR change. The results showed that a increase in CD8+ T cell count was an independent favorable prognostic factor for PFS (HR = 0.2308, 95%CI: 0.0875-0.5636, *p* = 0.002). Decreased NLR was associated with better PFS but did not reach statistical significance (HR = 0.4365, 95%CI: 0.1719-1.074, *p* = 0.073). Regarding lines of therapy, third-line or above treatment had an HR of 2.022 (95%CI: 0.7734-5.284, *p* = 0.151) compared with first-line. Among cancer types, hepatopancreatobiliary malignancies had an HR of 12.94 (95%CI: 2.260-63.29, *p* = 0.004) relative to lung cancer, indicating a very poor prognosis. CD4/CD8 change was not independently associated with PFS (HR = 0.6109, 95%CI: 0.2620-1.387, *p* = 0.237) (**Figure 4A**). Model diagnostics showed that the AIC of the null model (intercept only) was 266.2, and the AIC of the final model was 252.6, indicating improved model fit. Harrell’s C-index was 0.7949 (95%CI: 0.7291-0.8608), suggesting good discriminative ability.

### 3.5 Time-dependent ROC analysis

Time-dependent ROC analysis was performed based on the above multivariate Cox regression model. Due to the limited number of events (n=34), confidence intervals for the time-dependent AUCs were not estimated. The point estimates of AUC at 2.5 months, 3.5 months, and 5.7 months (the quartiles of PFS event times) were 0.727, 0.827, and 0.853, respectively, indicating good short- and medium-term predictive accuracy, with predictive ability improving over time (**Figure 4B**).

### 3.6 Risk stratification and prognostic prediction

#### 3.6.1 Risk score-based risk stratification model

A linear risk score formula was constructed based on the regression coefficients (β) from the multivariate Cox regression model, and the Risk Score was calculated.

**Figure 5:**
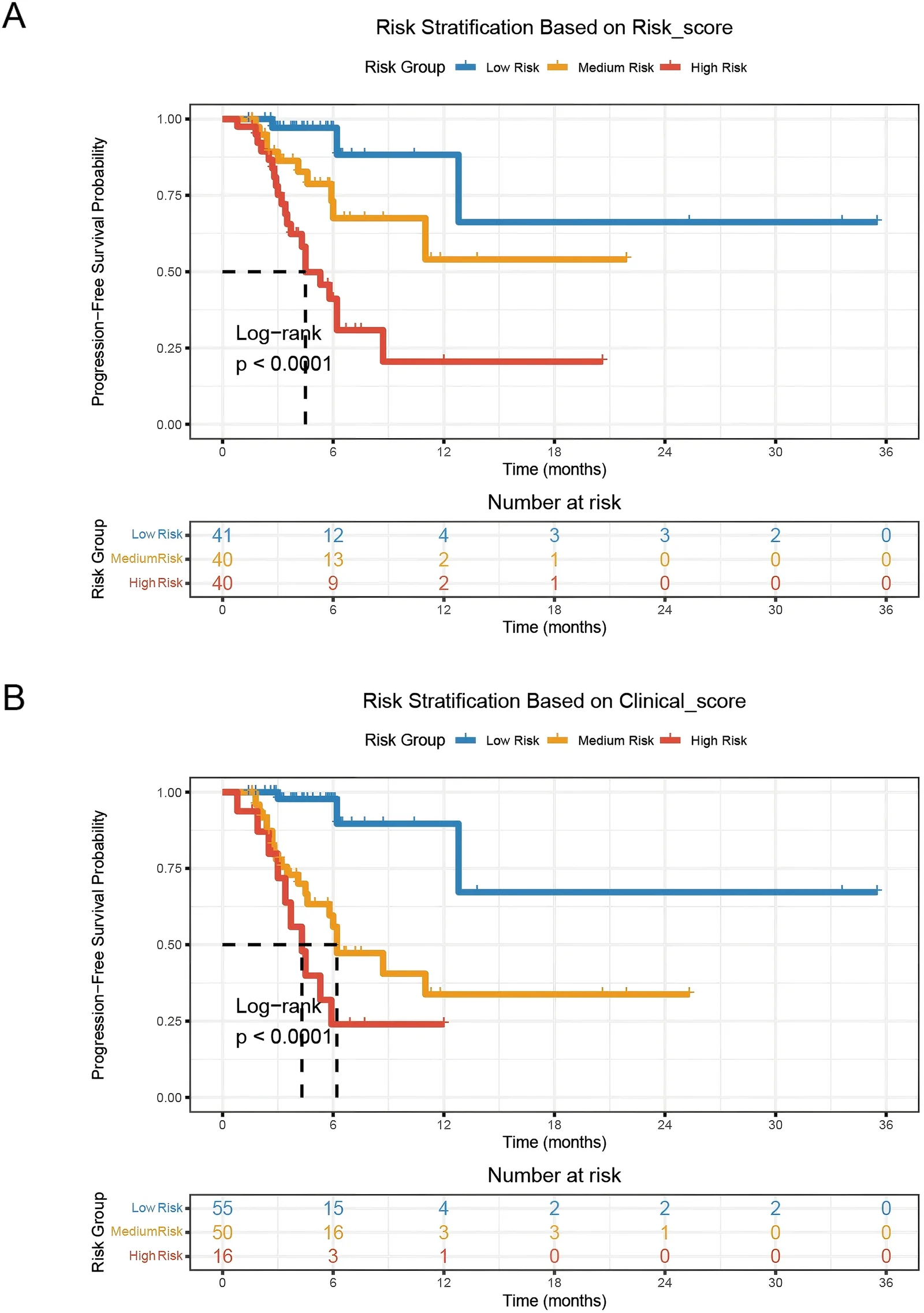
Kaplan-Meier curves for PFS stratified by Risk Score tertiles and by the simplified clinical scoring system. **(A)** Based on risk score, patients were divided into low-risk (Risk Score < -2.093, n=41), medium-risk (-2.093 to < -0.846, n=40), and high-risk (≥ -0.846, n=40) groups. Log-rank χ²=21.7, *p* = 2×10^-5^. **(B)** Based on the simplified clinical score (0–5 points), patients were grouped as low-risk (0-1, n=55), medium-risk (2-3, n=50), and high-risk (4-5, n=16). Log-rank χ²=25.9, *p* = 2×10^-6^. Tick marks indicate censored data. The number of patients at risk at each time point is shown below the X-axis.

Based on the tertiles of the Risk Score, the 121 patients were divided into three groups: low-risk (Risk Score < -2.093, n=41), medium-risk (-2.093 ≤ Risk Score < -0.846, n=40), and high-risk (Risk Score ≥ -0.846, n=40). The Kaplan-Meier survival curves showed highly significant differences in PFS among the three groups (log-rank χ²=21.7, *p* = 2×10⁻^5^) (**Figure 5A**). The median PFS was not reached in the low-risk group, not reached in the medium-risk group, and 4.5 months in the high-risk group. The 3-month PFS rates were 97.1%, 86.3%, and 75.2%; the 6-month PFS rates were 97.1%, 67.5%, and 41.1%; and the 12-month PFS rates were 88.3%, 54.0%, and 20.6%, respectively (**Table 4**).

**Table 4:** Risk stratification based on Risk Score and PFS outcomes.

Risk group	Risk score	N	Median PFS	3-month PFS rate	6-month PFS rate	12-month PFS rate
Low risk	< -2.093	41	Not reached	97.1% (91.8-100.0)	97.1% (91.8-100.0)	88.3% (72.6-100.0)
Medium risk	-2.093~ -0.846	40	Not reached	86.3% (75.8-98.3)	67.5% (51.0-89.4)	54.0% (32.1-90.9)
High risk	> -0.846	40	4.5 months	75.2% (62.3-90.7)	41.1% (26.1-64.7)	20.6% (7.5-56.1)

**Figure 6:**
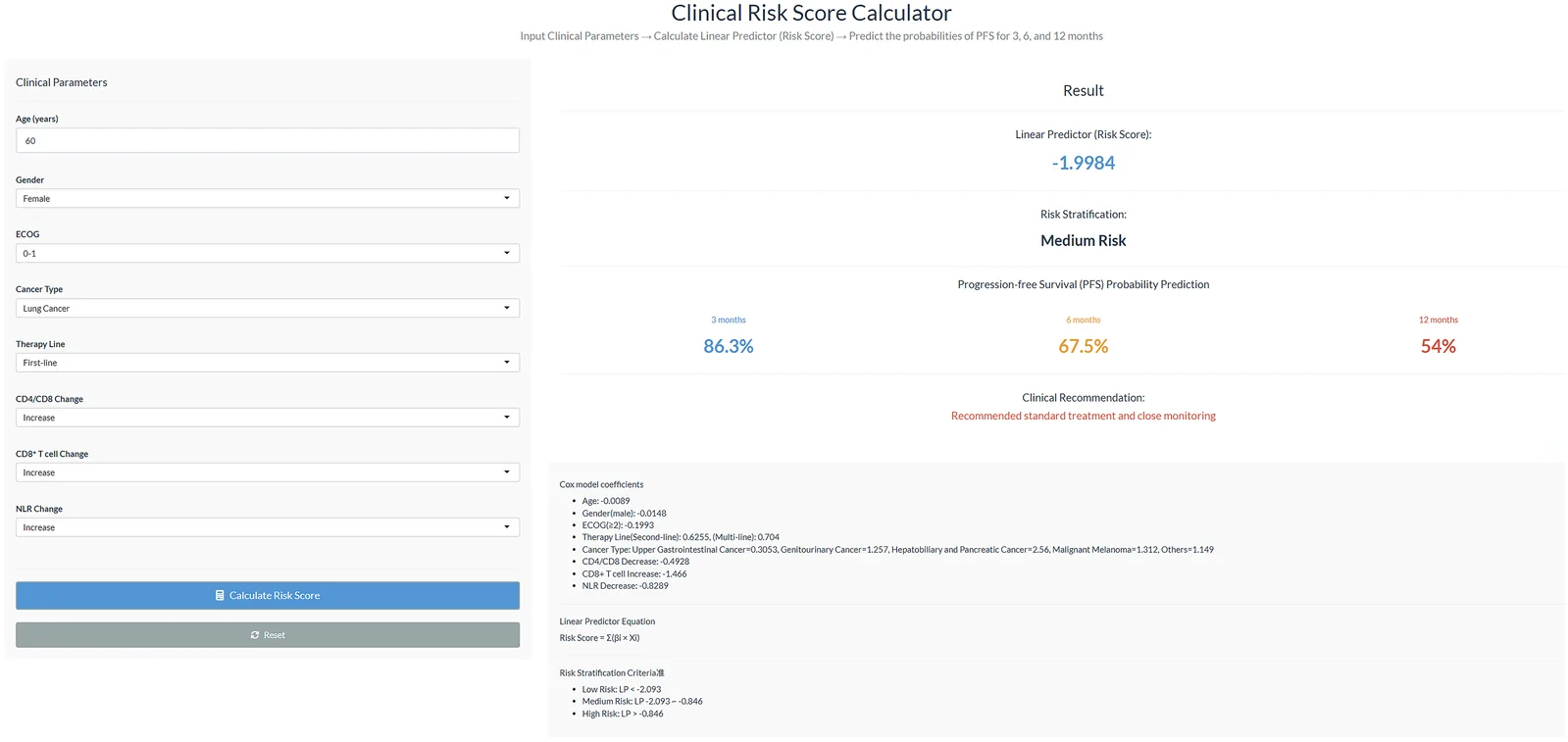
Screenshot of the web-based Risk Score calculator. This figure shows an interactive web-based calculator developed to provide individualized prediction of PFS for patients receiving ICI therapy. The calculator inputs include age, gender, CD4/CD8 change, CD8+ T cell change, NLR change, ECOG, line of therapy, and cancer type. Outputs include the Risk Score, risk stratification (low/medium/high), and clinical recommendations. Freely available at: *https://xmy13181.shinyapps.io/CALCULATOR/*. The figure shows the sample calculation results of a medium-risk patient. This screenshot is for demonstration purposes only. Please refer to the latest version of the webpage for actual use. This tool is for clinical research and reference in decision-making only and cannot replace the professional judgment of physicians.

Based on the above model, an interactive web-based calculator was developed at *https://xmy13181.shinyapps.io/CALCULATOR/*, where clinicians can input patient variables online to obtain the Risk Score and risk stratification in real time (**Figure 6**).

#### 3.6.2 Simplified clinical scoring system

To facilitate clinical application, we constructed a simplified scoring system based on clinically meaningful variables from the multivariate Cox regression. The assignment of points is shown in **Table 5**. The total score ranged from 0 to 5. According to the total score distribution, patients were divided into three groups: low-risk (0–1 points, n=55), medium-risk (2–3 points, n=50), and high-risk (4–5 points, n=16).

**Table 5:** Simplified clinical scoring system.

Variable	Condition	Points
CD8+ T cell Change	Increase	0
	Decrease	2
NLR Change	Decrease	0
	Increase	1
ECOG	0-1	0
	≥2	1
Therapy Line	First-line	0
	Second-line	1
	Third-line or above	2

Risk stratification based on total score:.

➢ Low risk: 0–1 points.

➢ Medium risk: 2–3 points.

➢ High risk: 4–5 points.

Kaplan-Meier survival analysis showed highly significant differences in PFS among the three groups (log-rank χ²=25.9, *p* = 2×10⁻^6^) (**Figure 5B**). The median PFS was not reached in the low-risk group, was 6.2 months in the medium-risk group, and was 4.3 months in the high-risk group. The 3-month PFS rates were 97.8%, 78.0%, and 71.8%; the 6-month PFS rates were 97.8%, 55.9%, and 23.9%; and the 12-month PFS rates were 89.6%, 33.8%, and 23.9%, respectively (**Table 6**).

**Table 6:** Risk stratification based on simplified clinical scoring system and PFS outcomes.

Risk group	Score	N	Median PFS	3-month PFS rate	6-month PFS rate	12-month PFS rate
Low risk	0-1	55	Not reached	97.8% (93.6-100.0)	97.8% (93.6-100.0)	89.6% (75.2-100.0)
Medium risk	2-3	50	6.2 months	78.0% (66.7-91.1)	55.9% (41.5-75.3)	33.8% (18.5-61.6)
High risk	4-5	16	4.3 months	71.8% (51.6-99.9)	23.9% (9.0-63.9)	23.9% (9.0-63.9)

#### 3.6.3 Nomogram

A nomogram was constructed based on a Cox regression model rebuilt after stepwise regression. The calibration curve showed considerable deviation between predicted probabilities and actual observed probabilities (**Supplementary Figure 4**), likely due to the limited sample size and small number of events (n=34). Therefore, the nomogram was not used as the primary prediction tool. We ultimately recommend the Risk Score calculator and the simplified clinical scoring system as practical tools for clinical prognostic assessment.

#### 3.6.4 Clinical decision recommendations

Based on the above risk stratification results, we propose the following clinical decision recommendations:

- Low-risk group (Risk Score < -2.093 or clinical score 0–1 points): Continue standard treatment with routine follow-up monitoring.
- Medium-risk group (-2.093 ≤ Risk Score < -0.846 or clinical score 2–3 points): Standard treatment plus close monitoring (e.g., shorter intervals for imaging re-evaluation, increased frequency of peripheral blood immune marker testing).
- High-risk group (Risk Score ≥ -0.846 or clinical score 4–5 points): Consider intensified treatment strategies or clinical trials.

## 4 Discussion

### 4.1 Main findings

This study systematically evaluated the dynamic changes of peripheral blood lymphocyte subsets in patients with malignancies receiving ICI therapy for treatment response monitoring and prognosis assessment. The main findings are: (1) The change in CD8+ T cell count before and after treatment is significantly associated with immunotherapy response and PFS, and this association is immunotherapy-specific; (2) The risk score model and simplified clinical scoring system based on CD8+ T cell change, NLR change, and clinical variables effectively stratified patient prognosis.

The core finding of this study is that the dynamic change in peripheral blood CD8+ T cell count before and after treatment is an independent predictor of PFS in patients receiving ICI therapy. In both the lung cancer subgroup and the pan-cancer cohort, patients with PD had a significant decrease in post-treatment CD8+ T cell count, while those with an increased CD8+ T cell count had significantly longer median PFS. This result is highly consistent with the biological function of CD8+ T cells as core effector cells of anti-tumor immunity. Effective immunotherapy should reverse the exhausted state of CD8+ T cells, promote their activation and proliferation, and thus manifest as increased or stable counts in peripheral blood (21–23). Conversely, a decrease in CD8+ T cell count may indicate a failure to reverse immunosuppressive signals or abnormal homing of CD8+ T cells to tumor tissues (24). Notably, the absence of an association between CD8+ T cell change and prognosis in the non-immunotherapy validation cohort strongly supports the value of this indicator as an immunotherapy-specific biomarker, ruling out the possibility that it is merely a phenomenon accompanying tumor burden reduction or general treatment response. However, the CD4/CD8 ratio change was significant in the lung cancer subgroup but not in the pan-cancer cohort. Several factors may explain this discrepancy. First, the proportion of first-line therapy was higher in the lung cancer subgroup, where the immune system is relatively intact, allowing the CD4/CD8 ratio to more faithfully reflect the true immune response. In contrast, the pan-cancer cohort included a larger number of patients receiving multi-line treatments, and prior therapies may have induced long-term remodeling of lymphocyte subsets, leading to abnormal baseline CD4/CD8 ratios and a loss of linear correlation between post-treatment changes and prognosis. Second, different cancer types possess distinct immunological features. Lung cancer, particularly non-small cell lung cancer, is generally considered a “hot” tumor, characterized by a high TMB, abundant CD8+ T cell infiltration, and strong immunogenicity. Consequently, changes in the CD4/CD8 ratio during treatment may more sensitively reflect the activation or suppression of anti-tumor immune responses. However, the pan-cancer cohort also included “cold” tumors such as hepatopancreatobiliary malignancies, which have sparse immune cell infiltration, thereby diluting the predictive value of CD4/CD8 ratio changes.

Based on the multivariate Cox regression model, we constructed two risk stratification tools. The Risk Score web-based calculator provides individualized risk scores based on precise regression coefficients, with AUCs at 2.5, 3.5, and 5.7 months of 0.727, 0.827, and 0.853, respectively, and a C-index of 0.7949, indicating good discriminative ability. The simplified clinical scoring system incorporates only CD8+ T cell change, NLR change, ECOG score, and line of therapy. The low-risk group (0–1 points) had a median PFS not reached, the medium-risk group (2–3 points) had a median PFS of 6.2 months, and the high-risk group (4–5 points) had a median PFS of only 4.3 months (log-rank *p* = 2×10⁻^6^). This simplified scoring system is particularly suitable for clinical application, allowing rapid bedside risk stratification to guide individualized treatment decisions.

Multivariate analysis showed that hepatopancreatobiliary malignancies had an HR of 12.94 (*p* = 0.004) compared with lung cancer, representing the cancer type with the worst prognosis in this cohort. This is consistent with the typical characteristics of “cold tumors” in this category: low mutational burden, low immune cell infiltration, and a highly immunosuppressive microenvironment. For these patients, ICI therapy alone has limited efficacy, and combination strategies such as chemotherapy, anti-angiogenic therapy, or agents targeting the TGF-β signaling pathway are more important (25). Emerging areas such as tumor neoantigen vaccines and drug screening based on tumor organoids and artificial intelligence large models may represent future directions of exploration (26–29).

### 4.2 Limitations

This study has several limitations that should be considered when interpreting the results.

#### 4.2.1 Sample size and statistical limitations

The sample size is relatively small (n=121) with a limited number of events (n=34), which restricts the statistical power of multivariate analyses and the reliability of subgroup analyses. Some variables (e.g., NLR change) had a *p*-value at the borderline level (0.073) in multivariate analysis and might reach statistical significance in a larger sample. Confidence intervals for time-dependent AUCs were not estimated due to insufficient events, weakening the robustness of the AUC estimates. So these point estimates should be interpreted with caution. The initially constructed nomogram showed poor calibration and was not used as the final tool, primarily due to model overfitting caused by the limited number of events.

#### 4.2.2 Lack of external validation

All data were collected retrospectively from a single center, and there is no external independent validation cohort. The generalizability and transportability of the model need to be further validated in multicenter prospective studies. Differences in the operation and quality control of lymphocyte subset testing across centers may also affect the comparability of results.

#### 4.2.3 Uncertainty regarding applicability after expansion of immunotherapy indications

The patients included in this study all received ICI monotherapy or combination therapy, and were mainly late-stage/metastatic patients treated in later lines. As the indications for ICIs continue to expand, the following questions remain to be answered:

- Patients without measurable lesions: For patients receiving adjuvant therapy or neoadjuvant therapy who achieve pathological complete response (pCR), conventional RECIST assessment is inapplicable. Whether dynamic changes in CD8+ T cells can serve as a monitoring indicator for such patients requires specifically designed studies.
- Neoadjuvant or conversion therapy: The use of ICIs in neoadjuvant therapy is increasing, but within the short time window of only a few weeks before and after treatment, whether changes in CD8+ T cells can accurately predict pathological response rates (MPR/pCR) lacks evidence-based support.
- Immunotherapy cross-line treatment: Some patients who progress on first-line ICI therapy may receive cross-line immunotherapy (e.g., switching to a different immune drug or combining with other agents). After the dual hits of prior immunotherapy and disease progression, the patient’s immune system undergoes profound remodeling (30), and whether the prognostic predictive value of CD8+ T cell baseline status and dynamic changes remains unchanged is unclear.
- Prediction of immune-related complications: ICI therapy can induce specific immunopathological reactions (31), which can be life-threatening in severe cases, and an important reason is the abnormal infiltration of immune cells (32, 33). Whether dynamic changes in CD8+ T cells or other lymphocyte subsets can serve as early warning indicators for irAEs deserves further exploration.

### 4.3 Future directions

Based on the findings and limitations of this study, future research can be pursued in the following directions.

First, multicenter, large-sample prospective cohort studies should be conducted to validate the predictive value of CD8+ T cell dynamics and the clinical utility of the risk stratification tools, and to establish standardized testing and quality control procedures.

Second, dedicated validation studies should be designed to address the unresolved questions regarding expanded indications: explore the relationship between CD8+ T cell changes and minimal residual disease (MRD) and long-term survival in adjuvant therapy patients without measurable lesions; evaluate the predictive value of early CD8+ T cell changes (2–4 weeks after treatment) for pathological response in neoadjuvant therapy cohorts; analyze whether the prognostic predictive performance of CD8+ T cells changes after immune system remodeling in cross-line immunotherapy cohorts; and establish prospective irAE monitoring cohorts to explore the association between changes in CD8+ T cells and other subsets and the risk and severity of irAEs.

Third, using multi-parameter flow cytometry or single-cell sequencing technologies, further dissect the subset composition and functional status of peripheral blood CD8+ T cells (e.g., central memory T cells, effector memory T cells, terminally differentiated effector T cells, exhausted phenotypes) (34–36), rather than focusing only on absolute count changes, to improve prediction precision.

Fourth, explore the combination of CD8+ T cell changes with other biomarkers (e.g., PD-1/PD-L1 expression, TMB, ctDNA dynamics) to build integrated prediction models for more accurate patient stratification.

## 5 Conclusion

The dynamic change in peripheral blood CD8+ T cell count before and after treatment is an independent predictor of PFS in patients receiving ICI therapy and exhibits immunotherapy specificity. A simplified risk stratification tool based on CD8+ T cell changes and clinical variables can effectively assess patient prognosis and inform individualized treatment decisions. Future multicenter prospective studies are needed to further validate the clinical value of this tool and to explore its potential application in expanded ICI scenarios (patients without measurable lesions, neoadjuvant therapy, cross-line immunotherapy) and its ability to predict immune-related adverse events.

## Data Availability

The raw data supporting the conclusions of this article will be made available by the authors, without undue reservation.

## References

[1] Gubin MM Zhang X Schuster H Caron E Ward JP Noguchi T . Checkpoint blockade cancer immunotherapy targets tumour-specific mutant antigens. Nature. (2014) 515:577–81. doi: 10.1038/nature13988 25428507 PMC4279952

[2] Dagher OK Schwab RD Brookens SK Posey AD Jr . Advances in cancer immunotherapies. Cell. (2023) 186:1814–1814.e1. doi: 10.1016/j.cell.2023.02.039 37059073

[3] Garassino MC Gadgeel S Speranza G Felip E Esteban E Dómine M . Pembrolizumab plus pemetrexed and platinum in nonsquamous non-small-cell lung cancer: 5-year outcomes from the phase 3 KEYNOTE-189 study. J Clin Oncol. (2023) 41:1992–8. doi: 10.1200/jco.22.01989 36809080 PMC10082311

[4] Hellmann MD Paz-Ares L Bernabe Caro R Zurawski B Kim SW Carcereny Costa E . Nivolumab plus ipilimumab in advanced non-small-cell lung cancer. N Engl J Med. (2019) 381:2020–31. doi: 10.1056/NEJMoa1910231 31562796

[5] Wolchok JD Chiarion-Sileni V Rutkowski P Cowey CL SChadendorf D Wagstaff J . Final, 10-year outcomes with nivolumab plus ipilimumab in advanced melanoma. N Engl J Med. (2025) 392:11–22. doi: 10.1056/NEJMoa2407417 39282897 PMC12080919

[6] Rini BI Plimack ER Stus V Gafanov R Hawkins R Nosov D . Pembrolizumab plus Axitinib versus Sunitinib for Advanced Renal-Cell Carcinoma. N Engl J Med. (2019) 380:1116–27. doi: 10.1056/NEJMoa1816714 30779529

[7] Burtness B Harrington KJ Greil R Soulières D Tahara M de Castro G Jr. . Pembrolizumab alone or with chemotherapy versus cetuximab with chemotherapy for recurrent or metastatic squamous cell carcinoma of the head and neck (KEYNOTE-048): a randomised, open-label, phase 3 study. Lancet. (2019) 394:1915–28. doi: 10.1016/s0140-6736(19)32591-7 31679945

[8] Chowell D Yoo SK Valero C Pastore A Krishna C Lee M . Improved prediction of immune checkpoint blockade efficacy across multiple cancer types. Nat Biotechnol. (2022) 40:499–506. doi: 10.1038/s41587-021-01070-8 34725502 PMC9363980

[9] Subbiah V Solit DB Chan TA Kurzrock R . The FDA approval of pembrolizumab for adult and pediatric patients with tumor mutational burden (TMB) ≥10: a decision centered on empowering patients and their physicians. Ann Oncol. (2020) 31:1115–8. doi: 10.1016/j.annonc.2020.07.002 32771306

[10] Havel JJ Chowell D Chan TA . The evolving landscape of biomarkers for checkpoint inhibitor immunotherapy. Nat Rev Cancer. (2019) 19:133–50. doi: 10.1038/s41568-019-0116-x 30755690 PMC6705396

[11] Das A Sudhaman S Morgenstern D Coblentz A Chung J Stone SC . Genomic predictors of response to PD-1 inhibition in children with germline DNA replication repair deficiency. Nat Med. (2022) 28:125–35. doi: 10.1038/s41591-021-01581-6 34992263 PMC8799468

[12] Adashek JJ Moran JA Le DT Kurzrock R . Lessons learned from a decade of immune checkpoint inhibition: The good, the bad, and the ugly. Cancer Metastasis Rev. (2025) 44:43. doi: 10.1007/s10555-025-10260-8 40183852 PMC11971148

[13] Waters C Halpenny D . iRECIST: A case based users guide for radiologists. Can Assoc Radiol J. (2026) 77:139–59. doi: 10.1177/08465371251355866 41546554

[14] Sharma P Goswami S Raychaudhuri D Siddiqui BA Singh P Nagarajan A . Immune checkpoint therapy-current perspectives and future directions. Cell. (2023) 186:1652–69. doi: 10.1016/j.cell.2023.03.006 37059068

[15] Derosiers N Aguilar W DeGaramo DA Posey AD Jr . Sweet immune checkpoint targets to enhance T cell therapy. J Immunol. (2022) 208:278–85. doi: 10.4049/jimmunol.2100706 35017217 PMC8830766

[16] Myers JA Miller JS . Exploring the NK cell platform for cancer immunotherapy. Nat Rev Clin Oncol. (2021) 18:85–100. doi: 10.1038/s41571-020-0426-7 32934330 PMC8316981

[17] Tanaka A Sakaguchi S . Regulatory T cells in cancer immunotherapy. Cell Res. (2017) 27:109–18. doi: 10.1038/cr.2016.151 27995907 PMC5223231

[18] Fiala O Tkadlecova M Kopecky J Hosek P Studentova H Vocka M . The prognostic role of baseline and early dynamics of peripheral blood cell ratios in metastatic renal cell carcinoma patients treated with nivolumab. In Vivo. (2026) 40:1065–79. doi: 10.21873/invivo.14261 41760301 PMC12949900

[19] Fang R Chen Y Huang B Wang Z Zhu X Liu D . Predicting response to PD-1 inhibitors in head and neck squamous cell carcinomas using peripheral blood inflammatory markers. Transl Oncol. (2025) 51:102222. doi: 10.1016/j.tranon.2024.102222 39616985 PMC11647630

[20] Lim ZF Wu X Zhu L Albandar H Hafez M Zhao C . Quantitative peripheral live single T-cell dynamic polyfunctionality profiling predicts lung cancer checkpoint immunotherapy treatment response and clinical outcomes. Transl Lung Cancer Res. (2024) 13:3323–43. doi: 10.21037/tlcr-24-260 39830778 PMC11736609

[21] Im SJ Hashimoto M Gerner MY Lee J Kissick HT Burger MC . Defining CD8+ T cells that provide the proliferative burst after PD-1 therapy. Nature. (2016) 537:417–21. doi: 10.1038/nature19330 27501248 PMC5297183

[22] Zhou L Velegraki M Wang Y Mandula JK Chang Y Liu W . Spatial and functional targeting of intratumoral Tregs reverses CD8+ T cell exhaustion and promotes cancer immunotherapy. J Clin Invest. (2024) 134:e180080. doi: 10.1172/jci180080 38787791 PMC11245154

[23] Dolina JS Van Braeckel-Budimir N Thomas GD Salek-Ardakani S . CD8(+) T cell exhaustion in cancer. Front Immunol. (2021) 12:715234. doi: 10.3389/fimmu.2021.715234 34354714 PMC8330547

[24] Bassez A Vos H Van Dyck L Floris G Arijs I Desmedt C . A single-cell map of intratumoral changes during anti-PD1 treatment of patients with breast cancer. Nat Med. (2021) 27:820–32. doi: 10.1038/s41591-021-01323-8 33958794

[25] Song F Hu B Liang XL Cheng JW Wang CG Wang PX . Anlotinib potentiates anti-PD1 immunotherapy via transferrin receptor-dependent CD8(+) T-cell infiltration in hepatocellular carcinoma. Clin Transl Med. (2024) 14:e1738. doi: 10.1002/ctm2.1738 39095323 PMC11296886

[26] Rojas LA Sethna Z Soares KC Olcese C Pang N Patterson E . Personalized RNA neoantigen vaccines stimulate T cells in pancreatic cancer. Nature. (2023) 618:144–50. doi: 10.1038/s41586-023-06063-y 37165196 PMC10171177

[27] Broutier L Mastrogiovanni G Verstegen MM Francies HE Gavarró LM Bradshaw CR . Human primary liver cancer-derived organoid cultures for disease modeling and drug screening. Nat Med. (2017) 23:1424–35. doi: 10.1038/nm.4438 29131160 PMC5722201

[28] Driehuis E Kretzschmar K Clevers H . Establishment of patient-derived cancer organoids for drug-screening applications. Nat Protoc. (2020) 15:3380–409. doi: 10.1038/s41596-020-0379-4 32929210

[29] Sarvepalli S Vadarevu S . Role of artificial intelligence in cancer drug discovery and development. Cancer Lett. (2025) 627:217821. doi: 10.1016/j.canlet.2025.217821 40414522

[30] Li J Wu C Hu H Qin G Wu X Bai F . Remodeling of the immune and stromal cell compartment by PD-1 blockade in mismatch repair-deficient colorectal cancer. Cancer Cell. (2023) 41:1152–1169.e7. doi: 10.1016/j.ccell.2023.04.011 37172580

[31] Damo M Hornick NI Venkat A William I Clulo K Venkatesan S . PD-1 maintains CD8 T cell tolerance towards cutaneous neoantigens. Nature. (2023) 619:151–9. doi: 10.1038/s41586-023-06217-y 37344588 PMC10989189

[32] Kunimasa K Isei T Nakamura H Kimura M Inoue T Tamiya M . Proliferative CD8(+) PD-1(+) T-cell infiltration in a pembrolizumab-induced cutaneous adverse reaction. Invest New Drugs. (2018) 36:1138–42. doi: 10.1007/s10637-018-0628-3 29947012

[33] Chen M Ma P Zhang Y Wang D Yu Z Fu Y . Divergent tumor and immune cell reprogramming underlying immunotherapy response and immune-related adverse events in lung squamous cell carcinoma. J Immunother Cancer. (2023) 11:e007305. doi: 10.1136/jitc-2023-007305 37857527 PMC10603341

[34] Xiao J Li Z Ding Y Zhu K Zheng Z Zhang Y . Uridine depletion impairs CD8^+^ T cell antitumor activity through N-glycosylation. Cell Metab. (2026) 38:616–632.e8. doi: 10.1016/j.cmet.2025.11.016 41468885

[35] Ma S Ong LT Jiang Z Lee WC Lee PL Yusuf M . Targeting P4HA1 promotes CD8(+) T cell progenitor expansion toward immune memory and systemic anti-tumor immunity. Cancer Cell. (2025) 43:213–231.e9. doi: 10.1016/j.ccell.2024.12.001 39729997

[36] van der Leun AM Traets JJH Vos JL Elbers JBW Patiwael S Qiao X . Dual immune checkpoint blockade induces analogous alterations in the dysfunctional CD8+ T-cell and activated treg compartment. Cancer Discov. (2023) 13:2212–27. doi: 10.1158/2159-8290.Cd-22-0851 37548431 PMC10551666

