# Supplementary material for: A predictive model for immunotherapy efficacy in cancer based on dynamic changes of CD8+ T cells: a pan-cancer retrospective study
Source: Front Immunol. 2026 Aug 10;17:1870840. doi: 10.3389/fimmu.2026.1870840 (PMC13500775; doi:10.3389/fimmu.2026.1870840)
Supplement: Supplementary file 1 [file Table1.docx]

**Supplementary Figure 1. Associations of pre-treatment lymphocyte subset counts/percentages with objective response in lung cancer subgroup (n=50).** (A-F) Bar graph comparing the initial percentages of CD3+ T cell, CD4+ T cell, CD8+ T cell, NK cell, B cell and Treg among response groups. (G-L) Bar graph comparing the initial counts of CD3+ T cell, CD4+ T cell, CD8+ T cell, NK cell, B cell and Treg among response groups. (M) Bar graph comparing the initial CD4/CD8 ratio among response groups. These parameters were not significantly associated with initial treatment response.

**Supplementary Figure 2. Associations of post-treatment lymphocyte subset counts/percentages with treatment response in lung cancer subgroup (n=50).** (A-F) Bar graph comparing the final percentages of CD3+ T cell, CD4+ T cell, CD8+ T cell, NK cell, B cell and Treg among response groups. (G-L) Bar graph comparing the final counts of CD3+ T cell, CD4+ T cell, CD8+ T cell, NK cell, B cell and Treg among response groups. (M) Bar graph comparing the final CD4/CD8 ratio among response groups. These parameters were not significantly associated with initial treatment response.

**Supplementary Figure 3. Associations of changes in lymphocyte subset counts/percentages with treatment response in lung cancer subgroup (n=50).** (A-F) Bar graph comparing the changes in percentages of CD3+ T cell, CD4+ T cell, CD8+ T cell, NK cell, B cell and Treg among response groups. (G-K) Bar graph comparing the changes in counts of CD3+ T cell, CD4+ T cell, NK cell, B cell and Treg among response groups. These parameters were not significantly associated with initial treatment response.

**Supplementary Figure 4. Associations of changes in peripheral blood CD4/CD8 ratio with treatment response and Kaplan-Meier curves for PFS according to the change in CD4/CD8 ratio and post-treatment NLR.** (A and B) Pan-cancer immunotherapy cohort (n=121): Waterfall plot showing individual patient changes in CD4/CD8 ratio. The change in CD4/CD8 ratio was not significantly associated with treatment response (CR/PR vs. PD, *p*=0.0405; SD vs. PD, *p*=0.1597). (C) Other cancer types (besides lung cancer) subgroups (n=71): The change in CD4/CD8 ratio was not significantly associated with treatment response (*p*=0.8958). (D) Pan-cancer cohort (n=121): CD4/CD8 ratio change; increase vs. decrease: *p*=0.1228. (E) Pan-cancer cohort (n=121): initial NLR; increase vs. decrease: *p*=0.1461. Tick marks indicate censored data. **p*<0.05, ***p*<0.01, ****p*<0.001.

**Supplementary Figure 5. Nomogram for predicting the probability of progression events and the corresponding calibration curve.** (A) Nomogram: This nomogram was constructed based on the stepwise Cox regression model to predict the probability of PFS in patients receiving ICI therapy. For each variable (cancer type, line of therapy, change in CD4/CD8 ratio, change in CD8+ T cell count), corresponding points are assigned. The total points (sum of individual points) are projected onto the risk axis at the bottom to obtain the predicted probability of PFS. (B) Calibration curve: This curve evaluates the agreement between the nomogram-predicted probabilities and the actual observed probabilities. The red dashed line indicates the ideal calibration line (predicted probability = actual probability). The blue solid line represents the calibration curve of the present model. The results show a substantial deviation of the blue line from the red dashed line, indicating poor calibration, likely due to overfitting caused by the limited sample size.
